# Targeting T-cell Aging to Remodel the Aging Immune System and Revitalize Geriatric Immunotherapy

**DOI:** 10.14336/AD.2025.0061

**Published:** 2025-03-12

**Authors:** Mi Chen, Zhou Su, Jianxin Xue

**Affiliations:** ^1^Division of Thoracic Tumor Multimodality Treatment, Cancer Center & State Key Laboratory of Biotherapy, West China Hospital, Sichuan University, Chengdu, Sichuan, China.; ^2^Department of Oncology, The Third Hospital of Mianyang, Sichuan Mental Health Center, Mianyang, Sichuan, China.; ^3^Department of Oncology, Mianyang 404 Hospital, Mianyang, Sichuan, China.; ^4^Laboratory of Clinical Cell Therapy, West China Hospital, Sichuan University, Chengdu, Sichuan, China.

**Keywords:** T cell aging, Aging immune system, Immunotherapy

## Abstract

The aging immune system presents profound challenges, notably through the decline of T cell function, which is critical for effective immune responses. As age-related changes lead to diminished T cell diversity and heighten immunosuppressive environments, older individuals face increased susceptibility to infections, autoimmune diseases, and reduced efficacy of immunotherapies. This review investigates the intricate mechanisms by which T cell aging drives immunosenescence, including immune suppression, immune evasion, reduced antigen reactivity, and the overexpression of immune checkpoint molecules. By delving into innovative therapeutic strategies aimed at rejuvenating T cell populations and modifying the immunological landscape, we highlight the potential for enhancing immune resilience in the elderly. Ultimately, our goal is to outline actionable pathways for restoring immune function, thereby improving health outcomes for aging individuals facing immunological decline.

## 1. T Cell Aging: A Barrier to Immunity and Immuno-therapy

Aging is the result of prolonged exposure to environmental stressors and intrinsic biological changes [1]. The aging process significantly impacts the immune system, leading to a decline in immune function and consequently weakening the body’s ability to respond effectively to new challenges [2]. As individuals age, their immune systems become increasingly vulnerable to a variety of diseases, including infections, autoimmune disorders, and malignancies [3]. This decline is characterized by alterations in both innate and adaptive immunity, with the adaptive immune system being particularly affected. The age-related loss of vitality in the adaptive immune system weakens immune responses, induces an immunosuppressive microenvironment, and leads to immune checkpoint blockade (ICB) and diminished immune defences [4]. This process is also accompanied by a reduction in the diversity of immune cell populations, leading to a diminished capacity to generate effective immune memory [5]. Moreover, the aging immune system contributes to reduced efficacy, or even loss, of immunotherapies such as cancer vaccines and CAR-T therapies, complicating the challenges of immunotherapy for elderly cancer patients [6, 7]. These manifestations of immune dysfunction not only heighten susceptibility to pathogens but also contribute to increased morbidity and mortality associated with aging [8].

T cells are crucial for coordinating immune responses. T cell aging represents a significant aspect of immune system decline, characterized by the gradual deterioration of T cell function due to prolonged immune stimulation and natural aging processes [9]. This decline manifests in older individuals as a reduced proliferative capacity, altered cytokine profiles, and impaired differentiation abilities of T cells [10]. Specifically, age-related immunological changes include a decrease in naïve T cells, an increase in memory T cells, reduced CD28 expression, and exacerbated T cell exhaustion characterized by elevated PD-1 expression [11]. Furthermore, the activity of antigen-presenting cells (APCs), such as dendritic cells and macrophages, is markedly diminished, while the activity of immunosuppressive cells, including regulatory T cells (Tregs) and myeloid-derived suppressor cells (MDSCs), is increased [4]. The accumulation of senescent T cells can create a pro-inflammatory environment, exacerbating age-related diseases [12]. Key factors contributing to T cell aging include thymic atrophy, mitochondrial dysfunction, genomic instability, reduced T cell receptor diversity, and decreased expression of co-stimulatory molecules [13]. These factors collectively impede the enhancement of immune capacity. Therefore, T cell aging is a critical component of immunosenescence, necessitating targeted interventions to restore immune function. Notably, Immunosenescence is the age-related decline in immune function, characterized by reduced adaptive immunity and increased inflammatory responses.

To address the challenges posed by T cell aging, it is essential to explore innovative strategies aimed at altering the phenotype of senescent T cells and reconstituting immune function. Therapeutic interventions targeting these age-related mechanisms present a promising approach [14]. Innovative strategies that restore the vitality of aged T cells, improve their metabolic adaptability, or modify the tumor microenvironment (TME) can enhance T cell functionality and extend the benefits of immunotherapy to the elderly population [11]. This review aims to provide a comprehensive discussion of current understanding regarding immune dysfunction during the aging process, elucidate the specific mechanisms underlying T cell aging, and introduce potential therapeutic avenues for reversing these age-related declines. Through this research, we aspire to establish a framework for improving immune health in the aging population, ultimately enhancing their ability to fight infection and disease.

## 2. The Hallmarks of T Cell Aging: Mechanisms of Development and Senescence

### 2.1 T-cell development and aging

T cell development commences in the bone marrow, where multipotent hematopoietic stem cells differentiate into common lymphoid progenitors, eventually leading to the formation of T lymphocytes [15]. This intricate process is critically influenced by the thymus, which serves as the primary site for T cell maturation [16]. Within the thymus, thymic epithelial cells (TECs) create dedicated microenvironments for T cell development and selection [17]. However, with advancing age, this developmental trajectory is significantly disrupted due to thymic involution, characterized by a reduction in thymic size and function, alongside a decline in lymphoid cell output from the bone marrow [18]. These age-related changes culminate in a marked decrease in the production of naïve T cells, resulting in an accumulation of highly differentiated T cell populations that exhibit a restricted T cell receptor (TCR) repertoire [16, 19]. In particular, the functional roles of γδ T cells evolve with age, demonstrating distinct profiles and tissue compartmentalization that influence overall immunity [19, 20]. This shift in T cell dynamics is a hallmark of immunosenescence, reflecting the complex relationship between aging and T cell development, including the role of age-specific signals required for thymic export and peripheral maturation of T cells [21].

T cell senescence is not merely a consequence of aging but a critical aspect of T cell development, characterized by a decline in functionality and the accumulation of intrinsic defects that impair immune responses [12]. As individuals age, naïve T cells undergo alterations that compromise their ability to respond effectively to new antigens, driven by changes in signaling pathways, reduced proliferative capacity, and altered cytokine production [22]. Interestingly, naïve CD4 T cells exhibit greater resistance to age-related loss than naïve CD8 T cells, suggesting CD4 T cell-specific protective mechanisms [23]. Another study reported an increase in naive CD8 T cells and a decrease in cytotoxic CD8 T cells and memory CD4 T cells in supercentenarians. This unique immune profile may contribute to their longevity [24].

Furthermore, the mechanisms behind these age-related changes are diversed, involving telomere shortening, increased oxidative stress, and the accumulation of senescence-associated secretory phenotypes (SASP). These factors foster a pro-inflammatory environment that limits effective immune responses and skews T cell repertoire diversity [25]. Profiling studies reveal an age-associated increase in type 2/IL-4-expressing memory T cell populations, indicating a shift in immune homeostasis [26]. Moreover, T cell-specific RIPK1 deficiency leads to premature senescence and various age-related diseases, with elevated mTORC1 activity driving increased cytokine production and senescence-related gene expression [27]. The interplay between these elements highlights the critical need to understand T cell senescence in the context of immunotherapy, as the age-associated decline in naïve T cell homeostasis poses significant challenges for effective therapeutic interventions in older populations [28].

### 2.2 Biological mechanisms of T cell aging

T cells are crucial for the immune system, helping to fight off infections and cancer. However, as people age, T cells can become senescent, meaning they lose their ability to divide and function effectively. This decline is influenced by several factors, including the aging of the thymus, the organ responsible for T cell development. As the thymus shrinks with age, fewer and less diverse T cells are produced, making it harder for the immune system to respond to new threats.

Mitochondrial dysfunction and genomic instability are key features of aging T cells. Older T cells often have damaged mitochondria, which affects their energy production and ability to activate properly. Tfam is a transcription factor crucial for mitochondrial biogenesis. Recent studies show that mice with B cell-specific Tfam deficiency exhibit a blockage in the germinal center (GC) reaction, associated with defects in lysosomal remodeling, and manifesting as an aged immune response [29]. Additionally, as T cells age, they accumulate genetic damage and mutations, which can impair their function. This instability, combined with the buildup of misfolded proteins due to disrupted protein balance, further weakens T cell responses.

Epigenetic changes, telomere shortening, and reduced lysosomal function also contribute to T cell aging, which leads to immunodeficiency [30, 31]. Changes in gene regulation can lead to a senescent state, while shorter telomeres limit how many times T cells can divide. Of note, some T cells can elongate telomeres by acquiring telomere vesicles from APCs, which allows them to remain protected from senescence before clonal division begins [32]. Furthermore, aging T cells struggle to clear out damaged components, which promotes their decline. As T cells become depleted, they exhibit diminished cytokine production and increased expression of inhibitory receptors, such as PD-1, CTLA-4, and LAG-3, along with the immunosuppressive enzyme CD39. This results in a reduced ability to mount effective immune responses.

### 2.3 Molecular features of T cell aging

T-cell senescence, driven by prolonged antigen exposure, leads to a decline of naive CCR7^+^ CD45RA^+^ T cells and an increase in terminally differentiated CCR7- CD45RO^+^ memory T cells, reshaping the immune landscape with age [33]. A significant feature of this process is the reduction of the TCR pool, which diminishes the diversity of responses to new antigens [13]. The increase in senescence-associated CD4 T cells that are refractory to TCR stimulation, contributing to the development of spontaneous germinal centers prone to autoantibody production [34]. Additionally, the loss of CD28 and CD27, crucial costimulatory receptors, is particularly pronounced in older individuals, with substantial proportions of CD4^+^ and CD8^+^ T cells exhibiting this deficiency [35]. This CD28-subset, along with upregulated markers such as CD57 and killer cell lectin-like receptor subfamily G member 1 (KLRG-1), indicates advanced differentiation stages and replicative senescence [7].

Senescent T cells also exhibit increased senescence-associated beta-galactosidase (SA-β-Gal) activity and the presence of γH2AX nuclear foci, indicating DNA damage [36]. Functionally, these senescent cells display defective mitochondria with low mitochondrial mass and increased reliance on glycolysis [37]. Moreover, they express elevated levels of SASP factors, such as interleukin (IL)-6, IL-8, and osteopontin, which contribute to chronic inflammation [38]. Notably, activated GZMK-expressing CD8 T cells, which accumulate with age, enhance the inflammatory functions of non-immune cells, highlighting their role in age-related dysfunctions of the immune system [39]. In addition to the functional impairments, age-related transcriptomic heterogeneity in immune cells indicates that frailty-a condition often associated with aging-results in distinct immune cell characteristics, including a frailty-specific monocyte subset exhibiting high expression of long noncoding RNAs NEAT1 and MALAT1 [40]. These findings suggest that such alterations contribute to the deterioration of immune status in frail individuals. These alterations culminate in diminished T-cell function, including impaired cytokine production and reduced proliferative potential, ultimately impacting immune response efficiency and contributing to age-related immunological challenges.

## 3 T-cell Aging as a Catalyst for Immune System Dysregulation

**Figure 1.**
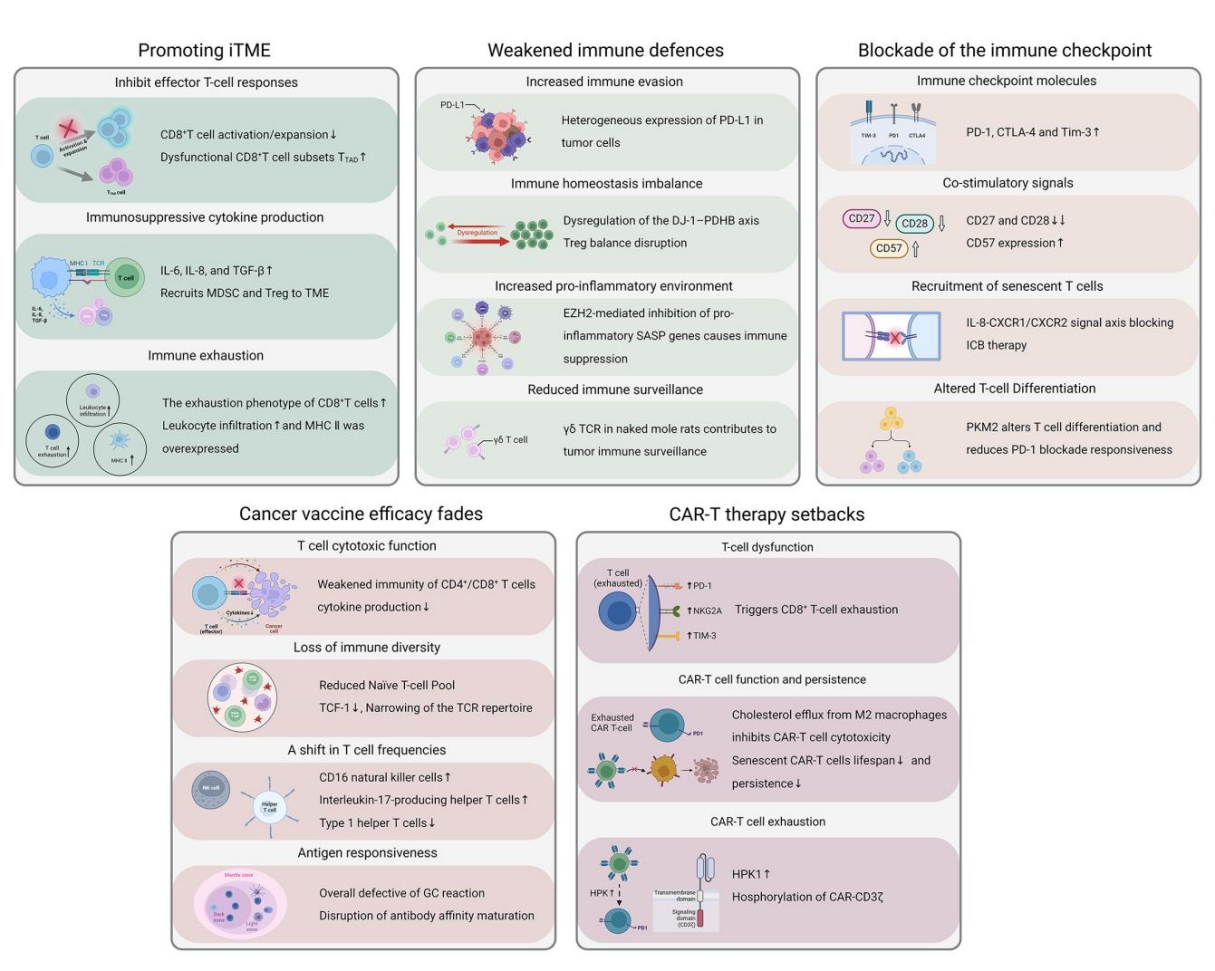
**The suppression of the immune system functions due to T cell senescence**. The aging immune system is affected by T cell senescence, which promotes the formation of an immunosuppressive tumor microenvironment, reduces immune defense, and obstructs immune checkpoints. Together, these factors contribute to a reduced response to immunotherapies in the elderly, including weakened or even failed responses to cancer vaccines and CAR-T therapies. iTME, immunosuppressive tumor microenvironment; MDSC, myeloid-derived suppressor cell; Treg, regulatory T cell; MHC Ⅱ, major histocompatibility complex Ⅱ; EZH2, enhancer of zeste homolog 2; SASP, senescence-associated secretory phenotype; TCR, T cell receptor; ICB, immune checkpoint blockade; PKM2, pyruvate kinase muscle 2; GC, germinal center; HPK1, Hematopoietic progenitor kinase 1.

As T cells age, they undergo various molecular and functional changes that can impair their ability to respond effectively to pathogens and therapies. The aging process contributes significantly to immune system dysregulation, leading to weakened immune defenses, the promotion of immunosuppressive microenvironments, and challenges in immunotherapy efficacy, including cancer vaccines and CAR-T therapies. Understanding these mechanisms is crucial for developing strategies to overcome the barriers posed by T cell aging. In this chapter, we will explore how T-cell aging catalyzes immune dysfunction, highlighting its role in the blockade of immune responses and the reduced efficacy of immunotherapies (Fig. 1).

### 3.1 Promoting immunosuppressive micro-environments

Rapid tumor growth is often accompanied by an immunosuppressive tumor microenvironment (iTME), which may limit the flexibility and resilience of T cell responses and is exacerbated by age-related immune deficits [41]. T-cell senescence severely alters the immune microenvironment, leading to impaired function and a diminished ability to eliminate tumor cells [42]. A recent study reveals that limited activation of CD8^+^ T cells in the TME of elderly individuals constrains tumor control more significantly than intracellular defects [43]. This research identifies a novel subset of dysfunctional T cells, known as T_TAD_ (tumor-infiltrating age-associated dysfunctional) cells, which are driven by extracellular signals and compromise anti-tumor immunity in older adults. Additionally, altered interactions among natural killer (NK) cells, dendritic cells, and CD8 T cells in aged tumors lead to impaired T cell activation in response to conventional type 1 dendritic cells, fostering the formation of T_TAD_ cells. Consequently, aged mice exhibit diminished responses to therapeutic tumor vaccines. Importantly, targeting myeloid cells can reactivate conventional type 1 dendritic cells, thereby enhancing tumor control and restoring CD8 T cell immunity in aged mice.

Senescent T cells adopt SASP, releasing factors that can recruit MDSCs and Tregs, creating an immunosuppressive environment that hinders effective anti-tumor responses [44]. For example, Genotoxic stress triggers DNA damage-induced senescence (DDIS) and SASP, which significantly reshape the TME by promoting inflammation and modifying immune responses [45]. JNK and Erk MAPK signaling pathways play a crucial role in initiating cellular senescence by responding to early DNA damage signals through the transcription factor AP-1 [45]. This disruption can reduce effector T cell populations, compromising the efficacy of immunotherapies such as checkpoint inhibitors.

Moreover, senescent T cells can induce senescence in neighboring effector T cells, perpetuating a cycle of immune dysfunction that facilitates tumor progression. Central nervous system-associated macrophages (CAMs) are crucial for coordinating the neuroimmune response and regulating adhesion molecules on endothelial cells. Age-related changes in CAMs lead to dysregulated immune responses, with their absence causing increased infiltration of CD4 and CD8 T lymphocytes, resulting in greater neurological dysfunction and an immunosuppressive environment [46]. CAMs also overexpress major histocompatibility complex class II (MHC II) to modulate these responses. Targeting CAMs is vital for preventing senescent T cell overactivity and effectively managing neuroimmune responses in aging. These findings underscore that senescent T cells and their interactions within the iTME not only limit effective immune responses but also contribute to a broader immunosuppressive landscape.

In a complementary study, antitumor CD8 T cell responses in young (prepubescent) versus adult (presenescent) mice demonstrate that young tumor-reactive CD8 T cells can become terminally differentiated, exhibiting overexpression of inhibitory receptors and the transcription factor Tox1 [47]. These terminally differentiated CD8 T cells show reduced cytokine responses, contributing to a less adaptable immune response. Moreover, young migratory dendritic cells (migDCs) and mononuclear phagocytic cells (MPCs) effectively capture and cross-present tumor antigens, promoting CD8 T cell priming and enhancing their terminal differentiation. Thus, T cell senescence reshapes the immune microenvironment. Addressing the challenges posed by T cell senescence is essential for enhancing the effectiveness of immunotherapeutic strategies and improving cancer treatment outcomes.

### 3.2 Weakened immune defences

T cell senescence significantly undermines immune surveillance, facilitating tumor progression and immune evasion [48]. PD-L1^+^ senescent cells accumulate with age and are more sensitive to T cell surveillance than PD-L1^-^ cells, which resist immune detection despite exhibiting SASP. Treatment with PD-1 antibodies in aging mice reduces the accumulation of p16 cells and PD-L1^+^ cells. Thus, targeting PD-L1 senescent cells by ICB may be an effective strategy for mitigating age-related issues [49].

As T cells age, they undergo metabolic reprogramming characterized by mitochondrial dysfunction and increased oxidative stress, which impair their proliferation and effector functions [50]. Key signaling pathways, such as mTOR and AMPK, become dysregulated, further compromising T cell responses to cancer [51]. An intriguing study reveals that the deglycase DJ-1 (PARK7) serves as a regulator of pyruvate dehydrogenase (PDH) activity in CD4 Tregs [52]. DJ-1 binds to PDHE1-β, inhibiting the phosphorylation of PDHE1-α, which enhances PDH activity and promotes oxidative phosphorylation. Dysregulation of the DJ-1-PDHB axis significantly disrupts Treg balance in aged mice, leading to impaired immune regulatory functions and exacerbating immune homeostasis imbalance due to T-cell senescence. These age-related changes not only diminish the ability of T cells to recognize and eliminate malignant cells but also create an environment conducive to cancer development. Targeting the molecular mechanisms underlying T cell senescence may offer innovative strategies to restore immune surveillance and enhance the effectiveness of immunotherapy in older patients [9].

The senescent T cell population is also influenced by a pro-inflammatory microenvironment, marked by chronic inflammation and the presence of SASP, which can inhibit T cell activation and promote tumor immune evasion [53]. Targeting SASP and inhibiting EZH2 may benefit older patients, particularly those with tumors such as pancreatic ductal adenocarcinoma (PDAC), by restoring immune surveillance [54]. The epigenetic repression of proinflammatory SASP genes mediated by EZH2 suppresses NK cell and T cell monitoring. Blocking EZH2 can enhance the production of chemokines such as CCL2 and CXCL9/10, promoting increased infiltration of NK and T cells, which can enhance the immunogenic binding of senescent cells and lead to tumor eradication. This strategy could convert immunologically "cold" tumors into "hot" tumors, thereby improving the efficacy of immunotherapy and providing better treatment outcomes for older patients facing age-related declines in immune function.

A promising study on the immune system of naked mole-rats may provide valuable insights [55]. G. Sanchez et al. discovered that the γδ T cells of naked mole-rats predominantly express a public invariant TCR, specifically the Vγ4-2/Vδ1-4 TCR, which includes distinct complementary-determining region 3 (CDR3) sequences likely generated through short-homology-repeat-driven DNA rearrangements. These invariant Vγ4-2/Vδ1-4 NK-like effector T cells play a crucial role in tumor immunosurveillance by mediating the recognition of common molecular signals associated with tumors through γδ TCR. For older patients experiencing declines in T cell function, leveraging or restoring similar γδ T cell mechanisms could rejuvenate or mimic their tumor recognition capabilities, ultimately enhancing immune responses in aging populations.

### 3.3 Blockade of the immune checkpoint

In aged individuals, the presence of senescent T cells can significantly dampen the efficacy of checkpoint blockade therapies [56]. With age, the ability of the immune system to mount robust anti-tumor responses is compromised by the accumulation of senescent T cells, characterized by reduced proliferative capacity, altered cytokine production and increased expression of inhibitory receptors [53]. This phenomenon is particularly relevant in the context of checkpoint inhibitors, which aim to reinvigorate exhausted T cells by blocking inhibitory pathways that tumors exploit to evade immune detection [57].

Senescent T cells often exhibit heightened expression of immune checkpoint molecules such as PD-1 and CTLA-4, which further inhibit T cell activation and proliferation.PD1 blockade has been shown to upregulate cytotoxic markers such as GzmB in CD8 T cells, increasing their cytotoxicity and protection against infection [58]. But even with checkpoint inhibition, the presence of a senescent T-cell population may limit the overall therapeutic response because of the reduced ability of these cells to respond to reactivation cues. This is because the loss of T-cell surface markers such as CD27 and CD28, or the expression of Tim-3 and CD57, leads to resistance to checkpoint inhibitor blockade [59]. Moreover, IL-8-CXCR1/CXCR2 signaling axis as a key player in immunosuppression within the TME, negatively impacting ICB efficacy [60]. While anti-PD-1 treatment can alleviate CD8 T cell exhaustion, it also increases systemic IL-8 levels and MDSC infiltration, further complicating treatment responses. This creates a paradox where the very mechanisms designed to enhance T cell activity may be undermined by the senescent state of a substantial portion of the T cell repertoire. The interplay between age-related immune checkpoint pathways suggests that a multifaceted approach may be necessary to enhance the efficacy of immunotherapy in older adults. A prospective study reports that targeting glycolysis through deletion of pyruvate kinase muscle 2 (PKM2) enhances the generation of TCF1high progenitor CD8 T cells with a progenitor-exhausted-like phenotype [57]. By metabolic reprogramming of T cells, the responsiveness to ICB therapy was enhanced.

### 3.4 Cancer vaccine efficacy fades

Studies indicate that vaccine efficacy in older adults is significantly lower than in younger individuals. Aging significantly impairs T-cell cytotoxic functionality through various molecular mechanisms, leading to reduced vaccine efficacy [61]. For example, the yearly influenza vaccine is only 40-60% effective in those aged 65 and older. Aging decreases S protein-specific IgG titers and CD4/CD8 T cell immunity, likely due to a reduced naive lymphocyte pool. This decline can lead to increased morbidity and mortality from vaccine-preventable diseases [62]. Senescence also delays antibody production and weakens CD8 T-cell responses, while older adults face impaired antigen processing and reduced T cell clonal expansion, contributing to a weaker immune response post-vaccination [63].

Increased levels of inhibitory receptors like PD-1 further suppress T-cell responses. Compared to younger adults, older adults have a reduced number of vaccine-induced spike-specific CD4^+^ T cells after receiving the first dose, including CXCR3^+^ circulating follicular helper T cells and TH1 subsets [64]. The inefficient CD4^+^ T cell response impairs T cell function, leading to fewer spike-specific CD4^+^ T cells and elevated levels of programmed cell death protein 1 (PD-1). The decreased activity of helper T cells results in reduced cytokine production, thereby lowering both humoral and cellular immunity.

Thymic involution decreases naive T cell production and disrupts the expression of essential transcription factors like TCF-1, resulting in a narrowed TCR repertoire [6]. Since cancer vaccines are designed to stimulate a naïve T-cell response, a reduced pool of naïve T cells in older adults means fewer T cells are available to respond to the tumor-specific antigens presented by the vaccine. Consequently, the immune system increasingly relies on memory T cells, which may not effectively respond to new antigens. Senescence also decreases vaccine efficacy in older adults through altered immune cell profiles, including increased cytotoxicity-associated gene expression and a shift in T cell frequencies, particularly affecting responses to the PCV13 vaccine [65]. A higher frequency of CD16 NK cells and IL-17-producing helper T cells was noted, while there was a decreased frequency of type 1 helper T cells. This shift can disrupt the balance of immune responses necessary for optimal vaccine efficacy.

The aging process also affects the molecular environment within lymphoid tissues, disrupting GC reactions vital for antibody affinity maturation, and thus affecting antigen responsiveness [61]. In older individuals, T follicular helper (TFH) cells mislocalize to the dark zone due to CXCR4-mediated mechanisms, leading to a compressed network of follicular dendritic cells (FDCs) in the light zone. This mislocalization hampers the quality of the antibody response; however, providing TFH cells that properly localize with FDCs can reverse these age-related defects, highlighting the importance of TFH cells in supporting effective immune responses to vaccines. Furthermore, senescence decreases vaccine efficacy by affecting the memory B cell response after influenza vaccination [66]. Older individuals show reduced expansion of hemagglutinin-specific B cells with an atypical FcRL5 phenotype, indicating diminished somatic hypermutation and positive selection within GCs. This leads to an overall defective GC reaction and impaired memory B cell response in older adults, contributing to their reduced vaccine effectiveness. These molecular alterations collectively contribute to the decline in vaccine efficacy in older adults. Targeting these mechanisms may enhance vaccine responses and improve immunotherapy strategies for this population.

### 3.5 CAR-T therapy setbacks

Currently, CAR-T therapy is revolutionizing cancer treatment [67]. By engineering a patient's T cells to express chimeric antigen receptors (CARs), these cells can effectively target and destroy tumor cells [68]. However, their ability to target only surface antigens poses limitations and faces challenges such as T cell exhaustion [69]. First, the patient's T cells are exposed to an iTME, triggering CD8 T cell exhaustion, which is a major obstacle to CAR-T cell therapy [70]. Second, cholesterol efflux from M2 macrophages can inhibit the cytotoxicity of CAR-T cells. This process places CD8 T cells in an immunosuppressive state, leading to exhaustion and a diminished response to immunotherapy [71]. Finally, the function, persistence, and exhaustion of CAR-T cells are also related to certain signaling pathways. Hematopoietic progenitor kinase 1 (HPK1) is a lesser-known kinase associated with LAT signaling during T cell activation, and high levels of HPK1 are linked to T cell exhaustion. Inhibition of HPK1 can enhance the efficacy of CAR-T cell therapy in preclinical models [72]. The phosphorylation of CAR-CD3ζ, driven by antigen-independent clustering of the CAR single-chain variable fragment, can induce early exhaustion of CAR-T cells. However, using the 4-1BB co-stimulation module instead of CD28 can mitigate this issue by recruiting the Themis-Shp1 complex to counteract the effects of Lck, thus reducing strong signaling and exhaustion [73]. In summary, the impairment of CAR-T cells due to exhaustion highlights that correcting tumor-associated T cell immunosenescence and exhaustion is crucial for enhancing the antitumor functions of engineered T cells.

## 4. Transforming T-cell Aging to Rejuvenate Immune Function

**Figure 2.**
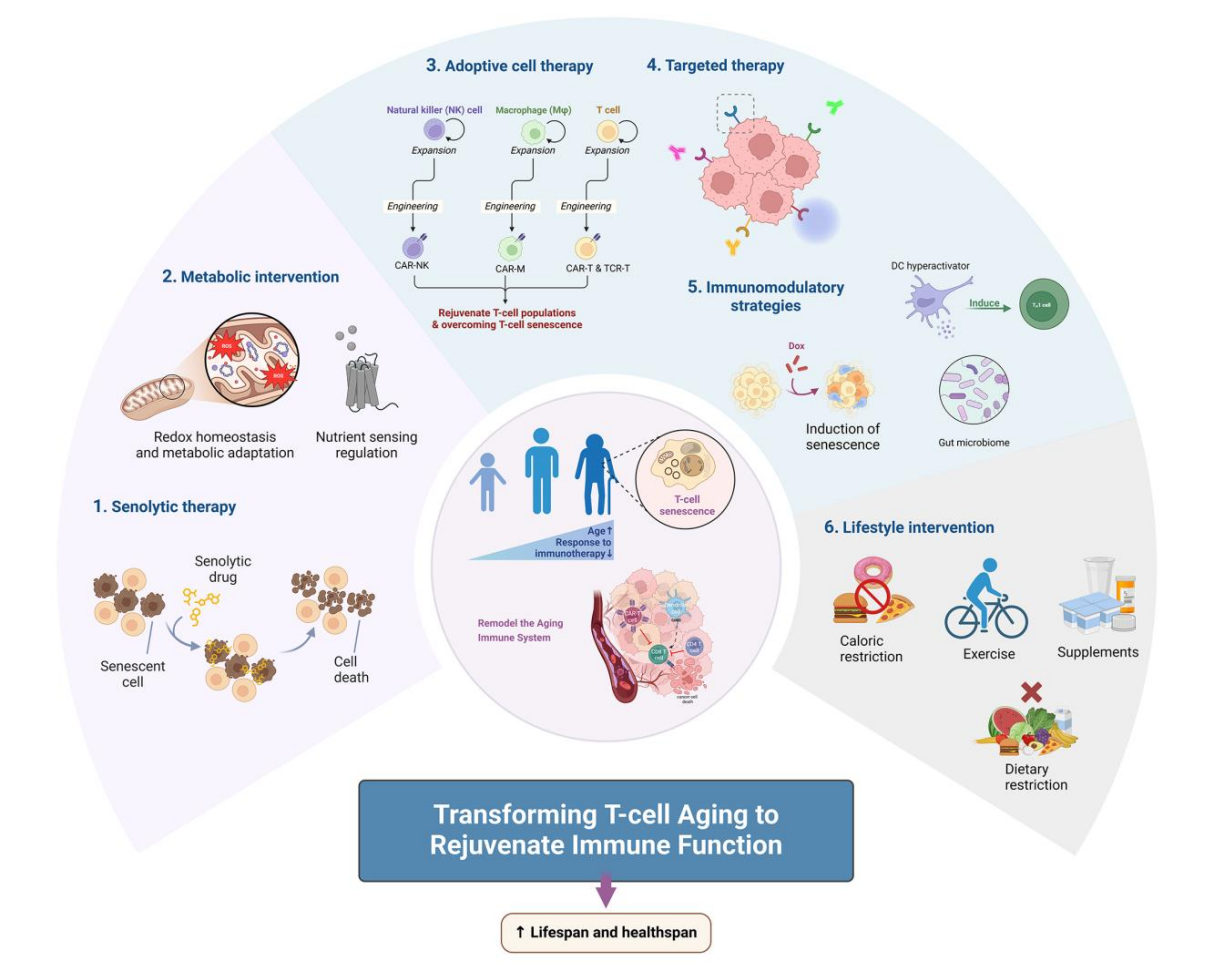
**A variety of strategies aimed at targeting T cell aging to rejuvenate the senescent immune system and enhance both lifespan and healthspan in the elderly**. The pie chart highlights six intervention tiers: (I) senolytic therapy aimed at eliminating senescent cells, (II) metabolic interventions to restore cellular function, (III) adoptive cell therapies to enhance immune responses, (IV) targeted therapies that focus on specific aging-related pathways, (V) innovative immunomodulatory strategies, and (VI) lifestyle interventions such as exercise and dietary modifications. DC, dendritic cell.

To counteract the negative effects of T-cell aging, numerous strategies have been proposed to rejuvenate immune function. These include senolytic therapies aimed at eliminating senescent cells, metabolic interventions to restore cellular function, adoptive cell therapies to enhance immune responses, and targeted therapies that focus on specific aging-related pathways. Additionally, innovative immunomodulatory strategies and lifestyle interventions, such as exercise and dietary modifications, are gaining attention for their potential to rejuvenate the immune system and improve therapeutic outcomes. This chapter will review these transformative approaches, focusing on their mechanisms and their promise in reversing the detrimental effects of T-cell aging on immunity (Fig. 2).

### 4.1 Senolytic therapy

To effectively delve into the promotion of anti-aging therapies in enhancing immunotherapy, several key studies highlight the beneficial effects of targeting T cell senescence [74, 75]. Research indicates that senescent cells play a pathogenic role in various diseases, and therapies aimed at clearing these cells, such as senolytic drugs, provide new avenues for improving immune function [76, 77]. For instance, dasatinib and quercetin, significantly reduce senescent cell populations, leading to decreased inflammation and improved immune function [78]. Similarly, metformin's ability to diminish CD4 T cell exhaustion post-vaccination suggests its potential in mitigating immune senescence [79]. Moreover, the interplay between gut microbiota and immune modulation has shown promise, with fecal microbial transplantation enhancing the efficacy of immune checkpoint inhibitors [80]. Ultimately, these interventions reveal the potential of anti-aging strategies in revitalizing T cell function and enhancing the overall effectiveness of immunotherapy.

A recent study showed that the varying expression of PD-L1 in senescent cells plays a crucial role in their immune evasion [49]. Specifically, PD-L1^+^ senescent cells are more susceptible to T cell attacks, while PD-L1^-^ cells are not. The use of PD-1 antibodies not only effectively reduces the number of these senescent cells but also elevated the cytotoxicity of CD8 CTLs, improving multiple aging-related conditions in mice and enhances immune responses. These findings provide the possibility for the combination of ICB and senolysis therapy. Further research into additional immune checkpoints could enhance these therapeutic approaches.

Another report clearly demonstrated that caraglipzin, by inhibiting SGLT2, was able to mitigate the accumulation of senescent cells linked to aging and metabolic stress [81]. SGLT2 inhibitors exhibit potential senolytic effects by enhancing AMPK activity. This action could restore immune function by reducing T cell anergy associated with senescence. Moreover, targeting PD-L1 in senescent cells may further enhance immune responses by activating endogenous senolysis mechanisms. Thus, combining SGLT2 inhibitors with immunotherapeutic strategies could ultimately rejuvenate T cell function and improve the efficacy of cancer treatments. To summarise, dual therapies combining senolytics and immunotherapy can target senescent and depleted cells to provide greater efficacy.

### 4.2 Metabolic intervention

The intricate relationship between aging and cancer highlights significant metabolic parallels that inform strategies to enhance T cell functionality in immunotherapy. Aged T cells often exhibit metabolic inflexibility, characterized by an impaired ability to switch between oxidative phosphorylation and glycolysis-an essential aspect of effective immune responses [82]. This dysfunction is exacerbated in the TME, where cancer cells exploit similar nutrient-sensing pathways, further compromising T cell efficacy. Specific signaling pathways, such as IL-7, promote branching in naive T cells, negatively impacting their function over time [83]. Additionally, as N-acetylglucosamine levels increase with age, this synergizes with IL-7, worsening T cell responses [84]. Changes in glycolysis also significantly affect CD8 T cell activation and function, contributing to their functional failure and recovery [85]. Recent studies reveal that the deglycase DJ-1 directly binds to PDH in CD4 Treg cells, stabilizing its active form by preventing oxidative inactivation. This interaction enhances PDH activity, facilitating the conversion of pyruvate to acetyl-CoA and promoting oxidative phosphorylation-a process critical for Treg survival and immunosuppressive function, especially in aged mice. The impairment of Treg functionality due to DJ-1 deletion underscores the connection between metabolic regulation and T cell homeostasis, emphasizing the DJ-1-PDHB axis as a potential therapeutic target [52]. The targeting of shared metabolic pathways, such as the modulation of IL-7, the manipulation of glycans, or the improvement of glycolysis, presents a compelling opportunity to rejuvenate T cell responses and enhance the effectiveness of immunotherapy in older patients.

The aging process significantly impacts T cell function through changes in mitochondrial bioenergetics and lipid metabolism, particularly due to the accumulation of the sphingolipid ceramide induced by aging stress [37]. Elevated C14/C16 ceramides in mitochondria of activated T cells from aging mice inhibit protein kinase A (PKA), leading to mitophagy and reduced antitumor capabilities. Therapeutic strategies to inhibit ceramide metabolism or activate PKA show promise in preventing mitophagy and restoring the central memory phenotype of aging T cells, suggesting that targeting ceramide-dependent pathways could enhance T cell antitumor activity in older patients [86]. More importantly, dysfunctional T cells lacking mitochondrial transcription factor A (TFAM) accelerate senescence and induce age-related features, contributing to chronic inflammation and premature aging, but interventions targeting cytokine signaling can partially mitigate these effects [87].

Both aging and cancer are marked by dysregulation of key nutrient-sensing pathways, particularly those involving insulin and IGF-1 signaling, mTOR (mechanistic target of rapamycin), and AMPK (AMP-activated protein kinase) [88]. These pathways play a crucial role in orchestrating cellular metabolism and energy homeostasis, directly influencing T cell activation, proliferation, and overall functionality. Recent findings highlight the crucial role of IL-11, a pro-inflammatory cytokine from the IL-6 family, in regulating the ERK-AMPK-mTORC1 axis as organisms age [89]. Elevated IL-11 levels in aging mice are linked to metabolic decline and multi-morbidity, with deletion of IL-11 or its receptor demonstrating protection against frailty. This inflammatory environment may contribute to the metabolic dysfunction observed in aged T cells, suggesting IL-11 as a potential therapeutic target. Meanwhile, new research shows that aging impairs asymmetric cell division (ACD) in CD8 T cells, leading to diminished expansion and memory potential [90]. Importantly, this impairment can be rescued through transient mTOR inhibition, suggesting a pivotal role for mTOR in maintaining T cell functionality in aging. Interestingly, the accumulation of T_VM_ cells during aging, which retain ACD capabilities and exhibit unique metabolic profiles, offers a glimmer of hope.

Recent findings regarding GIMAP5-a GTPase that regulates ceramide levels-underscore the significance of metabolic regulation in longevity and immune function [91]. GIMAP5's role in preventing the pathological accumulation of long-chain ceramides (CERs) is particularly relevant, as increased ceramide levels are associated with T cell senescence and dysfunction. By interacting with CK2 and attenuating its activation of ceramide synthases, GIMAP5 exemplifies how specific metabolic pathways can be targeted to restore T cell health. Consequently, targeting these shared metabolic routes, including GIMAP5, mTOR, and AMPK, presents compelling opportunities to rejuvenate T cell responses in older patients. Innovative therapeutic interventions that modulate these pathways could restore metabolic flexibility, enhance T cell activation, and improve the immune system's capacity to combat tumors. Ultimately, such strategies may lead to more effective immunotherapeutic regimens that harness the full potential of the immune response in older adults facing cancer, thereby improving clinical outcomes and quality of life.

### 4.3 Adoptive cell therapy

An increasing amount of evidence indicates that oncogene-driven senescent cells play a significant role in limiting the efficacy of cancer immunotherapy [92]. This senescence is characterized by a decline in T-cell functionality and an increase in exhaustion markers, ultimately compromising the immune response against tumors. Recent advancements in adoptive cell therapies, notably the use of CAR T cells, have shown promise in overcoming these challenges by reprogramming T cells to enhance their antitumor activity [93, 94]. The depletion of BATF enhances CAR T-cell performance by disrupting the AP-1/NFAT transcriptional axis, which normally promotes exhaustion-associated chromatin remodeling through histone deacetylase (HDAC) recruitment. This intervention shifts T-cell differentiation toward a central memory phenotype via upregulation of TCF7 and mitochondrial oxidative phosphorylation [93].

CAR T cells engineered to target senescence-associated proteins, such as the urokinase plasminogen activator receptor (uPAR) and NKG2D ligands, have demonstrated a remarkable ability to selectively eliminate senescent cells within the TME. This selective targeting not only improves overall immune responses but also ameliorates age-related metabolic dysfunctions observed in aged models, thus indicating a dual benefit of these therapies [76, 95, 96]. Moreover, small-molecule compounds like toosendanin have been identified as potential enhancers of CAR T-cell efficacy. It reprograms macrophages by inhibiting PI3K-γ, thereby reversing cAMP-mediated M2 polarization and enhancing phagocytic cross-presentation of tumor antigens. These compounds work by reprogramming macrophages, transforming them from immunosuppressive entities into active participants in the antitumor response, thereby mitigating the immunosuppressive effects driven by tumor-associated myeloid cells that complements CAR T-cell therapy [97].

The integration of NK cells with CAR technology represents another promising avenue in the fight against T-cell senescence. Studies have shown that NK cells can effectively target and eliminate stress-induced senescent cells, potentially reducing the levels of SASPs that contribute to systemic inflammation and further T-cell exhaustion [98]. CAR-NK therapies capitalize on NKG2D-mediated recognition of stress ligands (e.g., MICA/B) overexpressed on senescent cells, bypassing MHC restriction. Their efficacy is further potentiated by senolysis-induced reduction of SASP components like CCL2 and TGF-β. This approach not only enhances the overall immune landscape but also improves the potential for durable responses against tumors. The development of engineered artificial APCs that boost long-term T-cell memory also contributes to sustaining immune responses, reinforcing the multifaceted approaches needed to enhance the effectiveness of immunotherapy in clinic [99]. Together, these innovative strategies underscore the potential of adoptive cell therapies to rejuvenate T-cell populations, restore effective antitumor immunity, and ultimately improve therapeutic outcomes in aged populations facing age-related challenges in cancer treatment. By leveraging both CAR T-cell technology and NK cell therapies, we can address the multifaceted obstacles posed by T-cell senescence and significantly enhance the effectiveness of immunotherapy in the clinic.

### 4.4 Targeted therapy

Harnessing targeted therapies to combat the underlying factors of T-cell senescence could revolutionize immune rejuvenation in older adults. The aging immune system is characterized by a shift toward myeloid dominance, resulting in diminished lymphopoiesis and increased inflammation. Targeted depletion of myeloid-biased hematopoietic stem cells (my-HSCs) in aged mice enables balanced HSCs (bal-HSCs) to restore the hematopoietic system, enhancing lymphocyte progenitors and naive cells while reducing markers of lymphocyte dysfunction and inflammatory mediators [100].

Therapeutic strategies that target these dysfunctional T cells are gaining momentum, especially in cancer contexts where the TME can adversely affect immune responses [101]. The limited priming of CD8 T cells in aged environments is a significant barrier to effective tumor control, highlighting the urgent need for targeted interventions that can reinvigorate T cell function. Myeloid-targeted therapy aimed at enhancing the function of conventional type 1 dendritic cells in the aged TME to improve CD8 T cell priming and restore effective tumor control [43]. Age-related dysregulation significantly contributes to immunosenescence in CD8 T cells, which adopt an exhausted phenotype characterized by increased expression of inhibitory receptors, including PD1. While inhibiting PD1 has shown promise in enhancing CD8 T cell responses, it also carries risks of adverse immune reactions due to overactivation [58].

Innovative strategies targeting Tregs have surfaced. Recent studies reveal that Tregs play dual roles in immunosenescence: sustaining tissue repair while exacerbating immunosuppression. After thymic injury, circulating CD39^+^ICOS^+^ Tregs infiltrate the damaged thymus and secrete amphiregulin to promote stromal regeneration, suggesting their regenerative potential in aging-related thymic involution [102]. However, in elderly cancer patients, Tregs often adopt a pro-tumorigenic phenotype. For instance, SOAT2 overexpression in aged Tregs enhances cholesterol esterification, activating the SREBP2-HMGCR-GGPP pathway to amplify their suppressive function while impairing CD8^+^ T cell antitumor activity [103]. These findings highlight the metabolic plasticity of senescent Tregs. Long noncoding RNA Altre plays a critical role in regulating mitochondrial function and oxidative stress in Tregs during aging, greatly contributing to the maintenance of Treg function in the aging liver [104]. Importantly, the interplay between SOAT2-driven lipid metabolism and Altre-mediated mitochondrial homeostasis may coordinately dictate Treg functional fate. Hence, targeting Altre and its regulatory mechanisms could help mitigate immunosenescence and enhance antitumor immune responses, while combinatorial inhibition of SOAT2 may further reverse age-associated Treg hyper-suppression.

Furthermore, the regulation of CD8 T cell exhaustion is critical for sustained antitumor responses [82]. The identification of intratumor stem/progenitor-like CD8 T cell populations provides insights into mechanisms of T cell persistence and differentiation. The antagonistic roles of pathways involving BCL6 and BLIMP1 in regulating T cell fate underline the complexity of T cell dynamics in tumors and the potential for targeted interventions to enhance immunotherapy efficacy [105]. The identification of distinct T cell subsets during chronic infections also reveals potential vulnerabilities that can be targeted for therapeutic benefit. The transcription factors BATF and Tbx21 play key roles in CD8 T cell differentiation; therefore, targeting the regulation of these factors and their associated genes and epigenetic networks can effectively improve T cell function, helping to overcome T cell exhaustion in cancer [106].

According to a new study, infection-induced IL-33 production contributes to T cell aging and immunosuppression by causing thymic involution, which impairs the host's ability to manage severe infections [107]. IL-33 disrupts the balance between medullary thymic epithelial cells (mTECs) and cortical thymic epithelial cells (cTECs), leading to an excessive generation of mTEC IV (thymic tuft cells) and subsequent accumulation of mTEC I cells. This alteration in the thermic architecture hinders the development of naive T cells, resulting in diminished immune responses. Targeting IL-33 or its receptor ST2 may offer therapeutic strategies for rejuvenating T cell immunity. By addressing the multifaceted challenges posed by age-related immune dysfunction and leveraging targeted therapies, we can potentially ameliorate the effects of T-cell senescence on immunotherapy.

### 4.5 Innovative immunomodulatory strategies

The interplay between immune cell dynamics, particularly T cells and age-associated B cells (ABCs), is crucial in shaping the efficacy of cancer immunotherapies [108]. Recent studies highlight how T cell senescence and immune differentiation modulation can enhance therapeutic responses in aging populations. Defective host defenses in the elderly, such as diminished naive CD8 T cells and impaired dendritic cell (DC) migration, suggest that age-specific considerations are essential [109]. For instance, PD-1 and CTLA4-based immunotherapies show limited efficacy in elderly mice. Innovative approaches utilizing cellular senescence, like inducing senescence in breast cancer brain metastasis (BCBM) cells via doxorubicin, can enhance PD1-expressing T cell recruitment, improving anti-PD1 therapy efficacy [110]. Additionally, vaccine adjuvants, such as DC hyperactivators, can correct DC defects and induce cytolytic CD4 T cell responses, emphasizing the need to tailor immunotherapy strategies to age-related immune shifts [109]. The accumulation of age-associated T helper (T_H_A) cells and ABCs plays a pivotal role in autoimmunity and could impact cancer immunotherapy outcomes. Regulated by the transcription factor ZEB2, T_H_A cells demonstrate both cytotoxic activity and B cell helper functions. Furthermore, ZEB2 has been identified as a key driver of ABC formation. Therefore, targeting this transcription factor may provide a promising strategy for modulating immune responses in aging populations [111, 112].

The gut microbiota also plays a crucial role in tumor development and treatment response, especially in ICB therapy [113]. Research by Xiaoqiang Zhu et al. shows that enterotype-specific fecal microbiota transplantation (FMT) can significantly enhance mice's response to anti-PD-1 treatment. Customizing cancer immunotherapy based on individual gut microbiome characteristics can improve responsiveness to ICB therapy, thereby optimizing treatment outcomes for older patients [114]. Overall, by seeking to understand the underlying mechanisms of aging and immune cell interactions, researchers can develop tailored approaches to improve patient outcomes in the evolving landscape of immunotherapy.

### 4.6 Lifestyle intervention

Lifestyle interventions such as caloric restriction, exercise, supplements, and dietary restriction have shown promise in improving T-cell senescence and enhancing immune function. Caloric restriction has been linked to improved thymopoiesis and reduced inflammation by downregulating PLA2G7, while structured exercise significantly lowers markers of cellular senescence in T cells, suggesting enhanced immune responsiveness [115, 116]. Additionally, NAD^+^ restoration through supplementation may combat age-related declines in immune function, as its levels correlate with cellular health [117, 118]. Fasting induces a metabolic state that elevates ketone bodies, particularly β-hydroxybutyrate, which boosts the effector function of CD8^+^ T cells, thus enhancing their cytokine production and cytolytic activity [119]. Together, these interventions highlight a multifaceted approach to ameliorating T-cell senescence and improving immune efficacy.

### 4.7 Clinical trials

As shown in Table 1, several clinical trials are exploring strategies to address T cell aging and its impact on immune function, including a range of interventions aimed at rejuvenating the immune system. These strategies include senolytic therapies, such as fisetin, and metabolic interventions like somatropin, metformin, and DHEA, which target cellular senescence and metabolic pathways. Lifestyle changes, including physical exercise and fasting-mimicking diets, are also being investigated for their ability to modulate the immune system and combat aging-related declines. Some trials combine microbiota transplantation with ICB, such as anti-PD1 therapy, to improve immune responses, particularly in the context of cancer immunotherapy. Additionally, approaches to enhance immune responses to vaccination, such as supplementation with dairy protein and non-digestible polysaccharides, are being tested. Together, these trials reflect a growing interest in addressing T cell aging through a combination of pharmacological, metabolic, and lifestyle interventions, which may help restore immune function, improve vaccine efficacy, and overcome challenges in therapies like CAR-T. These strategies offer promising insights into combating immune system dysregulation associated with aging and enhancing the effectiveness of immunotherapies. Notably, despite promising clinical trial findings, several hurdles remain before these interventions can be widely implemented. Regulatory approval requires extensive safety and efficacy data, with challenges in defining appropriate biomarkers and endpoints for aging-related therapies. Additionally, variability in individual responses to interventions complicates trial design and real-world application. Ethical considerations, such as long-term risks and public acceptance, also need to be addressed to ensure responsible and equitable use of these therapies.

**Table 1:** Clinical studies and strategies for reverse immunosenescence registered in National Library of Medicine (NLM) at the United States National Institutes of Health (NIH).

NCT number	Target / Outcome Measure	Reversal strategy	Mode of administration	Status
**NCT06598839**	peripheral blood circulating tumor cells	Thymosin alpha 1	subcutaneous injection	Recruiting
**NCT04318964**	NY-ESO-1	TAEST16001 cell	intravenous injection	Active, not recruiting
**NCT04924374**	peripheral immune cells subpopulations and antitumoral immunity	Microbiota Transplant plus anti PD1 therapy	\	Completed
**NCT03178084**	CD4/CD8 Ratio	Zidovudine/lamivudine in combination with maraviroc or efavirenz	\	Completed
**NCT05421325**	innate immunity response	QBKPN SSI	subcutaneous injection	Recruiting
**NCT06431932**	senescent cells	Fisetin	oral treatment	Not yet recruiting
**NCT05940337**	monocytes	Nutrients	oral treatment	Completed
**NCT05190432**	immune System	Taxifolin, Ergothioneine	oral treatment	Active, not recruiting
**NCT01303484**	immune System	B-GOS	oral treatment	Completed
**NCT03557463**	immune response to vaccination	Dairy protein	oral treatment	Completed
**NCT01935271**	immune response to vaccination	Muscle armor supplement, Placebo	oral treatment	Completed
**NCT04375657**	epigenetic Age, assessment of naive T cells and immune cell function	Somatropin, metformin, and DHEA	\	Recruiting
**NCT04534049**	peripheral blood	Physical exercise	\	Unknown status
**NCT04928963**	immune response to vaccination, prevention of frailty	Fasting-mimicking Diet	\	Unknown status
**NCT05857241**	biological biochemic parameters	Therapeutic Fasting	\	Not yet recruiting
**NCT01896154**	immune response to vaccination	Non-digestible polysaccharides	oral treatment	Completed

## 5. Conclusions and perspectives

The intricate relationship between aging and T cell dysfunction underscores a critical challenge in enhancing immune resilience in the elderly. As highlighted in this review, the aging process profoundly alters T cell functionality, leading to an increased susceptibility to infections, reduced vaccine efficacy, and diminished responses to immunotherapies. Understanding the multifaceted mechanisms underlying T cell aging, from thymic involution and metabolic dysregulation to cellular senescence, offers valuable insights into potential therapeutic strategies. As our understanding of the indicators of T cell aging deepens, we can begin to rationalize therapeutic approaches aimed at halting or reversing age-related damage.

While significant strides have been made in elucidating the hallmarks of T cell aging, many questions remain regarding the interplay between these hallmarks and their cumulative impact on immune function. The dynamic nature of T cell senescence, coupled with the influence of the aging microenvironment, necessitates a more nuanced understanding of how these factors coalesce to perpetuate immune dysfunction. Specifically, the role of metabolic dysfunction as a central regulator of aging T cells invites further exploration. Targeting metabolic pathways may not only rejuvenate aged T cells but also restore their functional capabilities, paving the way for improved immunotherapeutic outcomes. Moreover, the heterogeneity of T cell populations, particularly in older individuals, calls for refined strategies that address the unique profiles of aging T cells [120]. The balance between maintaining a diverse repertoire and preventing oligoclonality is delicate; understanding how this balance shifts with age could reveal novel intervention points.

In advancing the field, there is a pressing need for innovative approaches that leverage current knowledge on cellular senescence and metabolic reprogramming. Strategies such as senolytic therapies to eliminate dysfunctional senescent T cells or metabolic modulators to enhance T cell efficacy hold promise. For adoptive cell therapies, challenges like the short in vivo persistence of allogeneic NK cells-requiring repeated infusions that heighten cytokine storm risks in immunosenescent hosts-must be addressed through engineering strategies to prolong cell survival. Furthermore, integrating high-dimensional technologies, such as single-cell transcriptomics, could illuminate the complexities of aging T cell subsets and their functional implications [121]. Concurrently, developing tissue-specific nanocarriers for drug delivery may mitigate off-target effects while enhancing therapeutic precision.

Looking ahead, it is imperative to bridge the gap between basic research and clinical applications. Aging-related immune decline varies significantly among individuals due to a combination of genetic predisposition, environmental influences, and comorbidities [122]. Genetic factors, such as polymorphisms in immune-regulatory genes, can influence T cell longevity, proliferative capacity, and response to senescence-associated signals [123]. Additionally, chronic conditions like diabetes, cardiovascular disease, and autoimmune disorders accelerate immunosenescence by inducing chronic inflammation and metabolic stress, further compromising immune resilience [124]. These variations in aging pathways contribute to heterogeneity in therapy effectiveness, underscoring the need for personalized approaches in immunomodulatory interventions.

The potential for geroprotective interventions to complement cancer immunotherapy in older adults warrants systematic investigation. Collaborative efforts between oncologists and geriatricians will be essential to tailor treatment strategies that account for both chronological and biological aging, ultimately enhancing the quality of life and therapeutic responses in the elderly. In conclusion, while the challenges posed by T cell aging are significant, they also present exciting opportunities for innovative therapies. Continued research will be crucial to unravel the complexities of T cell aging, allowing for the development of targeted interventions that can revitalize immune function in older populations and improve their ability to combat infections and diseases.

## References

[1] Lopez-OtinC, Blasco MA, PartridgeL, SerranoM, KroemerG (2023). Hallmarks of aging: An expanding universe. Cell, 186: 243-278.36599349 10.1016/j.cell.2022.11.001

[2] PhanHV, TsitsiklisA, MaguireC, HaddadE, BeckerP, Kim-SchulzeS, et al. (2024). Host-microbe multiomic profiling reveals age-dependent immune dysregulation associated with COVID-19 immunopathology. Sci Transl Med, 16: eadj5154.38630846 10.1126/scitranslmed.adj5154PMC11931290

[3] IskeJ, DedeiliaA, XiaoY, MartinF, EmmertMY, SagePT, et al. (2024). The impact of T-cell aging on alloimmunity and inflammaging. Transplantation, 108: 634-642.37389638 10.1097/TP.0000000000004715PMC10756935

[4] HanS, GeorgievP, Ringel AE, SharpeAH, HaigisMC (2023). Age-associated remodeling of T cell immunity and metabolism. Cell Metab, 35: 36-55.36473467 10.1016/j.cmet.2022.11.005PMC10799654

[5] AbbottA (2024). Hacking the immune system could slow ageing - here's how. Nature, 629: 276-278.38714810 10.1038/d41586-024-01274-3

[6] ChenJ, DengJC, GoldsteinDR (2022). How aging impacts vaccine efficacy: known molecular and cellular mechanisms and future directions. Trends Mol Med, 28: 1100-1111.36216643 10.1016/j.molmed.2022.09.008PMC9691569

[7] KasakovskiD, XuL, LiY (2018). T cell senescence and CAR-T cell exhaustion in hematological malignancies. J Hematol Oncol, 11: 91.29973238 10.1186/s13045-018-0629-xPMC6032767

[8] YousefzadehMJ, FloresRR, ZhuY, SchmiechenZC, BrooksRW, Trussoni CE, et al. (2021). An aged immune system drives senescence and ageing of solid organs. Nature, 594: 100-105.33981041 10.1038/s41586-021-03547-7PMC8684299

[9] Lopez-OtinC, PietrocolaF, Roiz-ValleD, GalluzziL, KroemerG (2023). Meta-hallmarks of aging and cancer. Cell Metab, 35: 12-35.36599298 10.1016/j.cmet.2022.11.001

[10] CarrascoE, Gomez de las HerasM M, Gabande-RodriguezE, Desdin-MicoG, Francisco ArandaJ, MittelbrunnM (2022). The role of T cells in age-related diseases. Nat Rev Immunol, 22: 97-111.34099898 10.1038/s41577-021-00557-4

[11] YuP-J, ZhouM, LiuY, DuJ (2024). Senescent T cells in age-related diseases. Aging Dis, 16: 321-44.38502582 10.14336/AD.2024.0219PMC11745454

[12] JinJ, MuY, ZhangH, SturmlechnerI, WangC, JadhavRR, et al. (2023). CISH impairs lysosomal function in activated T cells resulting in mitochondrial DNA release and inflammaging. Nature Aging, 3: 600-616.37118554 10.1038/s43587-023-00399-wPMC10388378

[13] MittelbrunnM, KroemerG (2021). Hallmarks of T cell aging. Nat Immunol, 22: 687-698.33986548 10.1038/s41590-021-00927-z

[14] MoqriM, HerzogC, PoganikJR, YingK, JusticeJN, Belsky DW, et al. (2024). Validation of biomarkers of aging. Nat Med, 30: 360-372.38355974 10.1038/s41591-023-02784-9PMC11090477

[15] HwangJ-R, ByeonY, KimD, ParkS-G (2020). Recent insights of T cell receptor-mediated signaling pathways for T cell activation and development. Exp Mol Med, 52: 750-761.32439954 10.1038/s12276-020-0435-8PMC7272404

[16] MaternaM, DelmonteOM, BosticardoM, MomenilandiM, ConreyPE, Charmeteau-De MuylderB, et al. (2024). The immunopathological landscape of human pre-TCR α deficiency: From rare to common variants. Science, 383: eadh4059.38422122 10.1126/science.adh4059PMC10958617

[17] RibeiroC, FerreirinhaP, Landry JJM, MacedoF, SousaLG, PintoR, et al. (2024). Foxo3 regulates cortical and medullary thymic epithelial cell homeostasis with implications in T cell development. Cell Death Dis, 15: 352.38773063 10.1038/s41419-024-06728-0PMC11109193

[18] Ramos SA, Armitage LH, Morton JJ, Alzofon N, Handler D, Kelly G, et al. (2023). Generation of functional thymic organoids from human pluripotent stem cells. Stem Cell Rep, 18: 829-840. 10.1016/j.stemcr.2023.02.013 PMC10147832 36963390

[19] GrayJI, CaronDP, WellsSB, GuyerR, SzaboP, RainbowD, et al. (2024). Human γδ T cells in diverse tissues exhibit site-specific maturation dynamics across the life span. Sci Immunol, 9: eadn3954.38848342 10.1126/sciimmunol.adn3954PMC11425769

[20] ChanS, MorganB, Yong MK, MargettsM, FarchioneAJ, LucasEC, et al. (2024). Cytomegalovirus drives Vdelta1+ gammadelta T cell expansion and clonality in common variable immunodeficiency. Nat Commun, 15: 4286-4286.38769332 10.1038/s41467-024-48527-3PMC11106253

[21] YangT, Barros-MartinsJ, WangZ, WenckerM, ZhangJ, SmoutJ, et al. (2023). RORγt+ c-Maf+Vγ4+ γδ T cells are generated in the adult thymus but do not reach the periphery. Cell Rep, 42: 113230.37815917 10.1016/j.celrep.2023.113230

[22] DurandA, BonillaN, LevelT, GinestetZ, LombesA, GuichardV, et al. (2024). Type 1 interferons and Foxo1 down-regulation play a key role in age-related T-cell exhaustion in mice. Nat Commun, 15: 1718.38409097 10.1038/s41467-024-45984-8PMC10897180

[23] CaoW, SturmlechnerI, ZhangH, JinJ, HuB, Jadhav RR, et al. (2023). TRIB2 safeguards naive T cell homeostasis during aging. Cell Rep, 42: 112195.36884349 10.1016/j.celrep.2023.112195PMC10118747

[24] ZhuH, ChenJ, LiuK, GaoL, WuH, MaL, et al. (2023). Human PBMC scRNA-seq-based aging clocks reveal ribosome to inflammation balance as a single-cell aging hallmark and super longevity. Sci Adv, 9: eabq7599.37379396 10.1126/sciadv.abq7599PMC10306289

[25] Xu Y, Wang Z, Li S, Su J, Gao L, Ou J, et al. (2024). An in-depth understanding of the role and mechanisms of T cells in immune organ aging and age-related diseases. Sci China Life Sci. 10.1007/s11427-024-2695-x 39231902

[26] TerekhovaM, SwainA, BohacovaP, AladyevaE, ArthurL, LahaA, et al. (2023). Single-cell atlas of healthy human blood unveils age-related loss of NKG2C+GZMB-CD8+memory T cells and accumulation of type 2 memory T cells. Immunity, 56: 2836-2854.37963457 10.1016/j.immuni.2023.10.013

[27] ImanishiT, UnnoM, YonedaN, MotomuraY, MochizukiM, SasakiT, et al. (2023). RIPK1 blocks T cell senescence mediated by RIPK3 and caspase-8. Sci Adv, 9: eadd6097.36696505 10.1126/sciadv.add6097PMC9876550

[28] WangL, ZhangX, ZhangH, LuK, LiM, LiX, et al. (2023). Excessive apoptosis of Rip1-deficient T cells leads to premature aging. Embo Rep, 24: e57925.37965894 10.15252/embr.202357925PMC10702839

[29] Iborra-PernichiM, Ruiz GarciaJ, Velasco de la EsperanzaM, Estrada BS, Bovolenta ER, CifuentesC, et al. (2024). Defective mitochondria remodelling in B cells leads to an aged immune response. Nat Commun, 15: 2569.38519473 10.1038/s41467-024-46763-1PMC10960012

[30] RodriguezRM, Saiz ML, Suarez-AlvarezB, Lopez-LarreaC (2022). Epigenetic networks driving T cell identity and plasticity during immunosenescence. Trends Genet, 38: 120-123.34561103 10.1016/j.tig.2021.08.014

[31] SchratzKE, FlaschDA, AtikCC, CosnerZL, BlackfordAL, YangW, et al. (2023). Report T cell immune deficiency rather than chromosome instability predisposes patients with short telomere syndromes to squamous cancers. Cancer Cell, 41: 807-817.37037617 10.1016/j.ccell.2023.03.005PMC10188244

[32] LannaA, VazB, D'AmbraC, ValvoS, VuottoC, ChiurchiuV, et al. (2022). An intercellular transfer of telomeres rescues T cells from senescence and promotes long-term immunological memory. Nat Cell Biol, 24: 1461-1474.36109671 10.1038/s41556-022-00991-zPMC7613731

[33] PanY-G, BartoloL, XuR, PatelBV, ZarnitsynaVI, SuLF (2024). Preservation of naive-phenotype CD4+T cells after vaccination contributes to durable immunity. Jci Insight, 9: e180667.38861490 10.1172/jci.insight.180667PMC11383171

[34] FukushimaY, SakamotoK, MatsudaM, YoshikaiY, YagitaH, KitamuraD, et al. (2022). cis interaction of CD153 with TCR/CD3 is crucial for the pathogenic activation of senescence-associated T cells. Cell Rep, 40: 111373.36130493 10.1016/j.celrep.2022.111373

[35] SlaetsH, VeeningenN, de KeizerP L J, HellingsN, HendrixS (2024). Are immunosenescent T cells really senescent? Aging Cell, 23: e14300.39113243 10.1111/acel.14300PMC11464117

[36] ZhaoH, LiuZ, ChenH, HanM, ZhangM, LiuK, et al. (2024). Identifying specific functional roles for senescence across cell types. Cell, 187: 7314-7334.39368477 10.1016/j.cell.2024.09.021

[37] AmorimJA, CoppotelliG, RoloAP, Palmeira CM, Ross JM, Sinclair DA (2022). Mitochondrial and metabolic dysfunction in ageing and age-related diseases. Nat Rev Endocrinol, 18: 243-258.35145250 10.1038/s41574-021-00626-7PMC9059418

[38] SuryadevaraV, HudginsAD, RajeshA, PappalardoA, KarpovaA, DeyAK, et al. (2024). SenNet recommendations for detecting senescent cells in different tissues. Nat Rev Mol Cell Bio, 25: 1001-1023.38831121 10.1038/s41580-024-00738-8PMC11578798

[39] MogilenkoDA, ShpynovO, AndheyPS, ArthurL, SwainA, EsaulovaE, et al. (2021). Comprehensive Profiling of an Aging Immune System Reveals Clonal GZMK+ CD8+ T Cells as Conserved Hallmark of Inflammaging. Immunity, 54: 99-115.33271118 10.1016/j.immuni.2020.11.005

[40] LuoOJ, LeiW, ZhuG, RenZ, XuY, XiaoC, et al. (2022). Multidimensional single-cell analysis of human peripheral blood reveals characteristic features of the immune system landscape in aging and frailty. Nature Aging, 2: 348-364.37117750 10.1038/s43587-022-00198-9

[41] GeorgievP, HanS, HuangAY, NguyenTH, Drijvers JM, CreaseyH, et al. (2024). Age-associated contraction of tumor-specific T cells impairs antitumor immunity. Cancer Immunol Res, 12: 1525-1541.39186561 10.1158/2326-6066.CIR-24-0463PMC11532741

[42] LiuX, Hoft DF, PengG (2020). Senescent T cells within suppressive tumor microenvironments: emerging target for tumor immunotherapy. J Clin Invest, 130: 1073-1083.32118585 10.1172/JCI133679PMC7269563

[43] ChenACY, JaiswalS, MartinezD, YerindeC, JiK, MirandaV, et al. (2024). The aged tumor microenvironment limits T cell control of cancer. Nat Immunol, 25: 1033-1045.38745085 10.1038/s41590-024-01828-7PMC11500459

[44] DambrosioM, GilJ (2023). Reshaping of the tumor microenvironment by cellular senescence: An opportunity for senotherapies. Dev Cell, 58: 1007-1021.37339603 10.1016/j.devcel.2023.05.010

[45] NetterfieldTS, OstheimerGJ, Tentner AR, Joughin BA, Dakoyannis AM, SharmaCD, et al. (2023). Biphasic JNK-Erk signaling separates the induction and maintenance of cell senescence after DNA damage induced by topoisomerase II inhibition. Cell Syst, 14: 582-604.37473730 10.1016/j.cels.2023.06.005PMC10627503

[46] LevardD, SeillierC, Bellemain-SagnardM, Fournier AP, LemarchandE, DembechC, et al. (2024). Central nervous system-associated macrophages modulate the immune response following stroke in aged mice. Nat Neurosci, 27: 1721-1733.38961228 10.1038/s41593-024-01695-3

[47] MoustakiA, CrawfordJC, AlliS, FanY, BoiS, Zamora AE, et al. (2022). Antigen cross-presentation in young tumor-bearing hosts promotes CD8+T cell terminal differentiation. Sci Immunol, 7: eabf6136.35119937 10.1126/sciimmunol.abf6136PMC8990347

[48] ZhangJ, HeT, XueL, GuoH (2021). Senescent T cells: a potential biomarker and target for cancer therapy. Ebiomedicine, 68: 103409.34049248 10.1016/j.ebiom.2021.103409PMC8170103

[49] WangT-W, JohmuraY, SuzukiN, OmoriS, MigitaT, YamaguchiK, et al. (2022). Blocking PD-L1-PD-1 improves senescence surveillance and ageing phenotypes. Nature, 611: 358-364.36323784 10.1038/s41586-022-05388-4

[50] WuH, ZhaoX, HochreinSM, EcksteinM, GubertGF, KnoepperK, et al. (2023). Mitochondrial dysfunction promotes the transition of precursor to terminally exhausted T cells through HIF-1α-mediated glycolytic reprogramming. Nat Commun, 14: 6858.37891230 10.1038/s41467-023-42634-3PMC10611730

[51] ChenY, XuZ, SunH, OuyangX, HanY, YuH, et al. (2023). Regulation of CD8+ T memory and exhaustion by the mTOR signals. Cell Mol Immunol, 20: 1023-1039.37582972 10.1038/s41423-023-01064-3PMC10468538

[52] DanileviciuteE, ZengN, CapelleCM, PacziaN, GillespieMA, KurniawanH, et al. (2022). PARK7/DJ-1 promotes pyruvate dehydrogenase activity and maintains Treg homeostasis during ageing. Nat Metab, 4: 589-607.35618940 10.1038/s42255-022-00576-y

[53] MarinI, BoixO, Garcia-GarijoA, SiroisI, CaballeA, ZarzuelaE, et al. (2023). Cellular Senescence Is Immunogenic and Promotes Antitumor Immunity. Cancer Discov, 13: 410-431.36302218 10.1158/2159-8290.CD-22-0523PMC7614152

[54] ChibayaL, MurphyKCC, DeMarco K DD, GopalanS, LiuH, ParikhCNN, et al. (2023). EZH2 inhibition remodels the inflammatory senescence-associated secretory phenotype to potentiate pancreatic cancer immune surveillance. Nat Cancer, 4: 872-892.37142692 10.1038/s43018-023-00553-8PMC10516132

[55] Sanchez SanchezG, EmmrichS, GeorgaM, PapadakiA, KossidaS, SeluanovA, et al. (2024). Invariant γδTCR natural killer-like effector T cells in the naked mole-rat. Nat Commun, 15: 4248.38762584 10.1038/s41467-024-48652-zPMC11102460

[56] LiuB, HuX, FengK, GaoR, XueZ, ZhangS, et al. (2022). Temporal single-cell tracing reveals clonal revival and expansion of precursor exhausted T cells during anti-PD-1 therapy in lung cancer. Nat Cancer, 3: 108-121.35121991 10.1038/s43018-021-00292-8

[57] MarkowitzGJ, BanY, TavarezDA, YoffeL, PodazaE, HeY, et al. (2024). Deficiency of metabolic regulator PKM2 activates the pentose phosphate pathway and generates TCF1+ progenitor CD8+ T cells to improve immunotherapy. Nat Immunol, 25: 1884-1899.39327500 10.1038/s41590-024-01963-1PMC12636053

[58] DahlquistKJV, HugginsMA, YousefzadehMJ, Soto-PalmaC, CholenskySH, PiersonM, et al. (2024). PD1 blockade improves survival and CD8+ cytotoxic capacity, without increasing inflammation, during normal microbial experience in old mice. Nature Aging, 4: 915-925.38689133 10.1038/s43587-024-00620-4PMC12142680

[59] MoreiraA, GrossS, KirchbergerMC, ErdmannM, SchulerG, HeinzerlingL (2019). Senescence markers: Predictive for response to checkpoint inhibitors. Int J Cancer, 144: 1147-1150.30151962 10.1002/ijc.31763

[60] LiuH, ZhaoQ, TanL, WuX, HuangR, ZuoY, et al. (2023). Neutralizing IL-8 potentiates immune checkpoint blockade efficacy for glioma. Cancer Cell, 41: 693-710.36963400 10.1016/j.ccell.2023.03.004

[61] Silva-CayetanoA, Fra-BidoS, RobertPA, InnocentinS, BurtonAR, WatsonEM, et al. (2023). Spatial dysregulation of T follicular helper cells impairs vaccine responses in aging. Nat Immunol, 24: 1124-1137.37217705 10.1038/s41590-023-01519-9PMC10307630

[62] DallanB, ProiettoD, De LaurentisM, GalleraniE, MartinoM, GhiselliniS, et al. (2024). Age differentially impacts adaptive immune responses induced by adenoviral versus mRNA vaccines against COVID-19. Nature Aging, 4: 1121-1136.38918602 10.1038/s43587-024-00644-w

[63] XiaoC, RenZ, ZhangB, MaoL, ZhuG, GaoL, et al. (2023). Insufficient epitope-specific T cell clones are responsible for impaired cellular immunity to inactivated SARS-CoV-2 vaccine in older adults. Nature Aging, 3: 418-435.37117789 10.1038/s43587-023-00379-0PMC10154213

[64] JoN, HidakaY, KikuchiO, FukahoriM, SawadaT, AokiM, et al. (2023). Impaired CD4+ T cell response in older adults is associated with reduced immunogenicity and reactogenicity of mRNA COVID-19 vaccination. Nature Aging, 3: 82-92.37118516 10.1038/s43587-022-00343-4PMC10154196

[65] RavichandranS, Erra-DiazF, KarakaslarOE, MarchesR, Kenyon-PesceL, RossiR, et al. (2024). Distinct baseline immune characteristics associated with responses to conjugated and unconjugated pneumococcal polysaccharide vaccines in older adults. Nat Immunol, 25: 316-329.38182669 10.1038/s41590-023-01717-5PMC10834365

[66] BurtonAR, GuillaumeSM, FosterWS, WheatleyAK, HillDL, Carr EJ, et al. (2022). The memory B cell response to influenza vaccination is impaired in older persons. Cell Rep, 41: 111613.36351385 10.1016/j.celrep.2022.111613PMC9666924

[67] WangX, WuX, TanB, ZhuL, ZhangY, LinL, et al. (2024). Allogeneic CD19-targeted CAR-T therapy in patients with severe myositis and systemic sclerosis. Cell, 187: 4890-4904.39013470 10.1016/j.cell.2024.06.027

[68] LarsonRC, MausMV (2021). Recent advances and discoveries in the mechanisms and functions of CAR T cells. Nat Rev Cancer, 21: 145-161.33483715 10.1038/s41568-020-00323-zPMC8353572

[69] XiongD, YuH, SunZ-J (2024). Unlocking T cell exhaustion: Insights and implications for CAR-T cell therapy. Acta Pharm Sin B, 14: 3416-3431.39220881 10.1016/j.apsb.2024.04.022PMC11365448

[70] Hossain MA, LiuG, DaiB, SiY, YangQ, WazirJ, et al. (2021). Reinvigorating exhausted CD8+ cytotoxic T lymphocytes in the tumor microenvironment and current strategies in cancer immunotherapy. Med Res Rev, 41: 156-201.32844499 10.1002/med.21727

[71] YanZ-X, DongY, QiaoN, ZhangY-L, WuW, ZhuY, et al. (2024). Cholesterol efflux from C1QB-expressing macrophages is associated with resistance to chimeric antigen receptor T cell therapy in primary refractory diffuse large B cell lymphoma. Nat Commun, 15: 5183.38890370 10.1038/s41467-024-49495-4PMC11189439

[72] SiJ, ShiX, SunS, ZouB, LiY, AnD, et al. (2020). Hematopoietic progenitor kinase1 (HPK1) mediates T cell dysfunction and is a druggable target for T cell-based immunotherapies. Cancer Cell, 38: 551-566.32860752 10.1016/j.ccell.2020.08.001

[73] SunC, ShouP, DuH, HirabayashiK, ChenY, Herring LE, et al. (2020). THEMIS-SHP1 recruitment by 4-1BB tunes LCK-mediated priming of chimeric antigen receptor-redirected t cells. Cancer Cell, 37: 216-225.32004441 10.1016/j.ccell.2019.12.014PMC7397569

[74] AmorC, FeuchtJ, LeiboldJ, HoY-J, ZhuC, Alonso-CurbeloD, et al. (2020). Senolytic CAR T cells reverse senescence-associated pathologies. Nature, 583: 127-132.32555459 10.1038/s41586-020-2403-9PMC7583560

[75] HasegawaT, OkaT, SonHG, Oliver-GarciaVS, AzinM, EisenhaureTM, et al. (2023). Cytotoxic CD4+ T cells eliminate senescent cells by targeting cytomegalovirus antigen. Cell, 186: 1417-1431.37001502 10.1016/j.cell.2023.02.033

[76] AmorC, Fernandez-MaestreI, ChowdhuryS, HoY-J, NadellaS, GrahamC, et al. (2024). Prophylactic and long-lasting efficacy of senolytic CAR T cells against age-related metabolic dysfunction. Nature Aging, 4: 336-349.38267706 10.1038/s43587-023-00560-5PMC10950785

[77] van DeursenJM (2019). Senolytic therapies for healthy longevity. Science, 364: 636-637.31097655 10.1126/science.aaw1299PMC6816502

[78] ChoiI, WangM, YooS, XuP, SeegobinSP, LiX, et al. (2023). Autophagy enables microglia to engage amyloid plaques and prevents microglial senescence. Nat Cell Biol, 25: 963-974.37231161 10.1038/s41556-023-01158-0PMC10950302

[79] YangJ, LiuH-C, ZhangJ-Q, ZouJ-Y, ZhangX, ChenW-M, et al. (2023). The effect of metformin on senescence of T lymphocytes. Immun Ageing, 20: 73.38087369 10.1186/s12979-023-00394-0PMC10714529

[80] KimY, KimG, KimS, ChoB, KimS-Y, DoE-J, et al. (2024). Fecal microbiota transplantation improves anti-PD-1 inhibitor efficacy in unresectable or metastatic solid cancers refractory to anti-PD-1 inhibitor. Cell Host Microbe, 32: 1380-1393.39059396 10.1016/j.chom.2024.06.010

[81] KatsuumiG, ShimizuI, SudaM, YoshidaY, FurihataT, JokiY, et al. (2024). SGLT2 inhibition eliminates senescent cells and alleviates pathological aging. Nature Aging, 4: 926-938.38816549 10.1038/s43587-024-00642-yPMC11257941

[82] FrancoF, JaccardA, RomeroP, YuY-R, HoP-C (2020). Metabolic and epigenetic regulation of T-cell exhaustion. Nat Metab, 2: 1001-1012.32958939 10.1038/s42255-020-00280-9

[83] WangC, KongL, KimS, LeeS, OhS, JoS, et al. (2022). The role of IL-7 and IL-7R in cancer pathophysiology and immunotherapy. Int J Mol Sci, 23: 10412.36142322 10.3390/ijms231810412PMC9499417

[84] MkhikianH, HayamaKL, KhachikyanK, LiC, Zhou RW, PawlingJ, et al. (2022). Age-associated impairment of T cell immunity is linked to sex-dimorphic elevation of N-glycan branching. Nature Aging, 2: 231-242.35528547 10.1038/s43587-022-00187-yPMC9075523

[85] CaoJ, LiaoS, ZengF, LiaoQ, LuoG, ZhouY (2023). Effects of altered glycolysis levels on CD8+ T cell activation and function. Cell Death Dis, 14: 407.37422501 10.1038/s41419-023-05937-3PMC10329707

[86] VaenaS, ChakrabortyP, LeeHG, JannehAH, Kassir MF, BeesonG, et al. (2021). Aging-dependent mitochondrial dysfunction mediated by ceramide signaling inhibits antitumor T cell response. Cell Rep, 35: 109076.33951438 10.1016/j.celrep.2021.109076PMC8127241

[87] Desdin-MicoG, Soto-HerederoG, Francisco ArandaJ, OllerJ, CarrascoE, Gabande-RodriguezE, et al. (2020). T cells with dysfunctional mitochondria induce multimorbidity and premature senescence. Science, 368: 1371-1376.32439659 10.1126/science.aax0860PMC7616968

[88] van VlietT, Varela-EirinM, WangB, BorghesanM, Brandenburg SM, FranzinR, et al. (2021). Physiological hypoxia restrains the senescence-associated secretory phenotype via AMPK-mediated mTOR suppression. Mol Cell, 81: 2041-2052.33823141 10.1016/j.molcel.2021.03.018

[89] WidjajaAA, LimW-W, ViswanathanS, ChothaniS, CordenB, DasanCM, et al. (2024). Inhibition of IL-11 signalling extends mammalian healthspan and lifespan. Nature, 632: 157-165.39020175 10.1038/s41586-024-07701-9PMC11291288

[90] BorsaM, BarandunN, GraebnitzF, BarnstorfI, BaumannNS, PallmerK, et al. (2021). Asymmetric cell division shapes naive and virtual memory T-cell immunity during ageing. Nat Commun, 12: 2715.33976157 10.1038/s41467-021-22954-yPMC8113513

[91] ParkAY, Leney-GreeneM, LynbergM, Gabrielski JQ, XuX, SchwarzB, et al. (2024). GIMAP5 deficiency reveals a mammalian ceramide-driven longevity assurance pathway. Nat Immunol, 25: 282-293.38172257 10.1038/s41590-023-01691-yPMC11151279

[92] SchmittCA, WangB, DemariaM (2022). Senescence and cancer - role and therapeutic opportunities. Nat Rev Clin Oncol, 19: 619-636.36045302 10.1038/s41571-022-00668-4PMC9428886

[93] ZhangX, ZhangC, QiaoM, ChengC, TangN, LuS, et al. (2022). Depletion of BATF in CAR-T cells enhances antitumor activity by inducing resistance against exhaustion and formation of central memory cells. Cancer Cell, 40: 1407-1422.36240777 10.1016/j.ccell.2022.09.013

[94] KawaiY, Kawana-TachikawaA, KitayamaS, UedaT, MikiS, WatanabeA, et al. (2021). Generation of highly proliferative, rejuvenated cytotoxic T cell clones through pluripotency reprogramming for adoptive immunotherapy. Mol Ther, 29: 3027-3041.34023508 10.1016/j.ymthe.2021.05.016PMC8530944

[95] DengY, KumarA, XieK, SchaafK, ScifoE, MorsyS, et al. (2024). Targeting senescent cells with NKG2D-CAR T cells. Cell Death Discov, 10: 217.38704364 10.1038/s41420-024-01976-7PMC11069534

[96] YangD, SunB, LiS, WeiW, LiuX, CuiX, et al. (2023). NKG2D-CAR T cells eliminate senescent cells in aged mice and nonhuman primates. Sci Transl Med, 15: eadd1951.37585504 10.1126/scitranslmed.add1951

[97] YangF, ZhangD, JiangH, YeJ, ZhangL, BagleySJ, et al. (2023). Small-molecule toosendanin reverses macrophage-mediated immunosuppression to overcome glioblastoma resistance to immunotherapy. Sci Transl Med, 15: eabq3558.36791206 10.1126/scitranslmed.abq3558PMC10394757

[98] BaiZ, YangP, YuF, LiZ, YaoZ, MartinezJ, et al. (2022). Combining adoptive NK cell infusion with a dopamine-releasing peptide reduces senescent cells in aged mice. Cell Death Dis, 13: 305.35383143 10.1038/s41419-022-04562-wPMC8983684

[99] Hasani-SadrabadiMM, MajediFS, ZarubovaJ, Thauland TJ, ArumugaswamiV, HsiaiTK, et al. (2024). Harnessing biomaterials to amplify immunity in aged mice through T memory stem cells. Acs Nano, 18: 6908-6926.38381620 10.1021/acsnano.3c08559

[100] RossJB, MyersLM, NohJJ, Collins MM, CarmodyAB, MesserRJ, et al. (2024). Depleting myeloid-biased haematopoietic stem cells rejuvenates aged immunity. Nature, 628: 162-170.38538791 10.1038/s41586-024-07238-xPMC11870232

[101] LuJ-C, WuL-L, SunY-N, HuangX-Y, GaoC, GuoX-J, et al. (2024). Macro CD5L+ deteriorates CD8+T cells exhaustion and impairs combination of Gemcitabine-Oxaliplatin-Lenvatinib-anti-PD1 therapy in intrahepatic cholangiocarcinoma. Nat Commun, 15: 621.38245530 10.1038/s41467-024-44795-1PMC10799889

[102] LemarquisAL, KousaAI, ArgyropoulosKV, JahnL, GipsonB, PierceJ, et al. (2025). Recirculating regulatory T cells mediate thymic regeneration through amphiregulin following damage. Immunity, 58:397-411.e6.39892391 10.1016/j.immuni.2025.01.006PMC11932356

[103] ZhangM, CuiJ, ChenH, ChengY, ChenQ, ZongF, et al. (2025). Increased SOAT2 expression in aged regulatory T cells is associated with altered cholesterol metabolism and reduced anti-tumor immunity. Nat Commun, 16: 630.39805872 10.1038/s41467-025-56002-wPMC11729894

[104] DingC, YuZ, SefikE, ZhouJ, KaffeE, WangG, et al. (2023). A Treg-specific long noncoding RNA maintains immune-metabolic homeostasis in aging liver. Nature Aging, 3: 813-828.37277640 10.1038/s43587-023-00428-8

[105] SunQ, CaiD, LiuD, ZhaoX, LiR, XuW, et al. (2023). BCL6 promotes a stem-like CD8+ T cell program in cancer via antagonizing BLIMP1. Sci Immunol, 8: eadh1306.37862431 10.1126/sciimmunol.adh1306

[106] ChenY, ZanderRA, WuX, Schauder DM, Kasmani MY, ShenJ, et al. (2021). BATF regulates progenitor to cytolytic effector CD8+ T cell transition during chronic viral infection. Nat Immunol, 22: 996-1007.34282329 10.1038/s41590-021-00965-7PMC9258987

[107] XuL, WeiC, ChenY, WuY, ShouX, ChenW, et al. (2022). IL-33 induces thymic involution-associated naive T cell aging and impairs host control of severe infection. Nat Commun, 13: 6881.36371464 10.1038/s41467-022-34660-4PMC9653498

[108] CuiC, WangJ, FagerbergE, ChenP-M, ConnollyKA, DamoM, et al. (2021). Neoantigen-driven B cell and CD4 T follicular helper cell collaboration promotes anti-tumor CD8 T cell responses. Cell, 184: 6101-6118.34852236 10.1016/j.cell.2021.11.007PMC8671355

[109] ZhivakiD, Kennedy SN, ParkJ, BorielloF, DevantP, CaoA, et al. (2024). Correction of age-associated defects in dendritic cells enables CD4+T cells to eradicate tumors. Cell, 187: 3888-3903.38870946 10.1016/j.cell.2024.05.026PMC11283364

[110] Uceda-CastroR, MargaridoAS, CornetL, VegnaS, HahnK, SongJ-Y, et al. (2022). Re-purposing the pro-senescence properties of doxorubicin to introduce immunotherapy in breast cancer brain metastasis. Cell Rep Med, 3: 100821.36384097 10.1016/j.xcrm.2022.100821PMC9729880

[111] DaiD, GuS, HanX, DingH, JiangY, ZhangX, et al. (2024). The transcription factor ZEB2 drives the formation of age-associated B cells. Science, 383: 413-421.38271512 10.1126/science.adf8531PMC7616037

[112] GotoM, TakahashiH, YoshidaR, ItamiyaT, NakanoM, NagafuchiY, et al. (2024). Age-associated CD4+ T cells with B cell-promoting functions are regulated by ZEB2 in autoimmunity. Sci Immunol, 9: eadk1643.38330141 10.1126/sciimmunol.adk1643

[113] LiuL, ShahK (2022). The Potential of the Gut Microbiome to Reshape the Cancer Therapy Paradigm. Jama Oncol, 8: 1059-1067.35482355 10.1001/jamaoncol.2022.0494

[114] ZhuX, HuangX, HuM, SunR, LiJ, WangH, et al. (2024). A specific enterotype derived from gut microbiome of older individuals enables favorable responses to immune checkpoint blockade therapy. Cell Host Microbe, 32: 489-505.38513657 10.1016/j.chom.2024.03.002

[115] SpadaroO, YoumY, ShchukinaI, RyuS, SidorovS, RavussinA, et al. (2022). Caloric restriction in humans reveals immunometabolic regulators of health span. Science, 375: 671-677.35143297 10.1126/science.abg7292PMC10061495

[116] EnglundDA, SakamotoAE, FritscheCM, HeerenAA, ZhangX, KotajarviBR, et al. (2021). Exercise reduces circulating biomarkers of cellular senescence in humans. Aging Cell, 20: e13415.34101960 10.1111/acel.13415PMC8282238

[117] SundaramB, PandianN, KimHJ, AbdelaalHM, MallR, IndariO, et al. (2024). NLRC5 senses NAD plus plus depletion, forming a PANoptosome and driving PANoptosis and inflammation. Cell, 187: 4061-4077.38878777 10.1016/j.cell.2024.05.034PMC11283362

[118] Covarrubias AJ, PerroneR, GrozioA, VerdinE (2021). NAD+ metabolism and its roles in cellular processes during ageing. Nat Rev Mol Cell Bio, 22: 119-141.33353981 10.1038/s41580-020-00313-xPMC7963035

[119] LudaKM, LongoJ, Kitchen-GoosenSM, DuimstraLR, MaEH, WatsonMJ, et al. (2023). Ketolysis drives CD8+T cell effector function through effects on histone acetylation. Immunity, 56: 2021-2035.37516105 10.1016/j.immuni.2023.07.002PMC10528215

[120] Fang F, Cao W, Zhu W, Lam N, Li L, Gaddam S, et al. (2021). The cell-surface 5’-nucleotidase CD73 defines a functional T memory cell subset that declines with age. Cell Rep, 37. 10.1016/j.celrep.2021.109981 PMC8612175 34758299

[121] WengN-P (2023). Transcriptome-based measurement of CD8+T cell age and its applications. Trends Immunol, 44: 542-550.37248098 10.1016/j.it.2023.05.005PMC10330598

[122] NogalskaA, EerdengJ, AkreS, Vergel-RodriguezM, LeeY, BramlettC, et al. (2024). Age-associated imbalance in immune cell regeneration varies across individuals and arises from a distinct subset of stem cells. Cell Mol Immunol, 21: 1459-1473.39443746 10.1038/s41423-024-01225-yPMC11607082

[123] SchmiedelBJ, SinghD, MadrigalA, Valdovino-GonzalezAG, WhiteBM, et al.Zapardiel-Gonzalo J (2018). Impact of Genetic Polymorphisms on Human Immune Cell Gene Expression. Cell, 175:1701-1715.e16.30449622 10.1016/j.cell.2018.10.022PMC6289654

[124] RohmTV, MeierDT, OlefskyJM, DonathMY, (2022). Inflammation in obesity, diabetes, and related disorders. Immunity, 55: 31-55.35021057 10.1016/j.immuni.2021.12.013PMC8773457

